# Radiation-Induced Heart Diseases: Protective Effects of Natural Products

**DOI:** 10.3390/medicina55050126

**Published:** 2019-05-09

**Authors:** Ahmed Eleojo Musa, Dheyauldeen Shabeeb

**Affiliations:** 1 Department of Medical Physics, Tehran University of Medical Sciences (TUMS), International Campus, Tehran 1416753955, Iran; 2 Research Center for Molecular and Cellular Imaging, TUMS, Tehran 1416753955, Iran; 3 Department of Physiology, College of Medicine, University of Misan, Misan 62010, Iraq; zahamtop5@gmail.com

**Keywords:** ionizing radiation, heart disease, radiotherapy, natural products, cancer, inflammation

## Abstract

Cardiovascular diseases (CVDs) account for the majority of deaths worldwide. Radiation-induced heart diseases (RIHD) is one of the side effects following exposure to ionizing radiation (IR). Exposure could be from various forms such as diagnostic imaging, radiotherapy for cancer treatment, as well as nuclear disasters and nuclear accidents. RIHD is mostly observed after radiotherapy for thoracic malignancies, especially left breast cancer. RIHD may affect the supply of blood to heart muscles, leading to an increase in the risk of heart attacks to irradiated persons. Due to its dose-limiting consequence, RIHD has a negative effect on the therapeutic efficacy of radiotherapy. Several methods have been proposed for protection against RIHD. In this paper, we review the use of natural products, which have shown promising results for protection against RIHD.

## 1. Introduction

Cardiovascular diseases (CVDs) are the leading causes of mortality worldwide [1]. They include all heart and circulatory diseases such as heart attack, angina, coronary heart disease, stroke, congenital heart disease, hypertension, and vascular dementia. Nowadays, humans are exposed to ionizing radiation (IR) in various forms, including diagnostic imaging and therapeutic purposes. While diagnostic imaging accounts for most radiation exposure to humans [2,3,4,5], more than half of cancer patients make use of IR during their course of treatment (a process known as radiation therapy or radiotherapy) [6].

One of the side effects following exposure to IR is radiation-induced heart disease (RIHD). RIHD could appear in the form of coronary artery disease, pericarditis, cardiomyopathy, and myocardial fibrosis, pericardial effusions or constriction, ischemia, valvular disease, arrhythmias, etc. [7,8]. Although, these complications could arise in patients undergoing radiotherapy for various malignancies in the thoracic and mediastinal regions, such as lung cancer, a large portion has been observed in left breast cancer patients irradiated at the chest [9]. This is unsurprising considering the close proximity between the left breast and the heart. Studies have also shown an increased risk of myocardial infarction (MI) after radiotherapy of left breast cancer compared to the right [10,11]. The dose-limiting consequences of these complications can negatively affect the therapeutic efficacy of radiotherapy for thoracic malignancies [12,13].

The incidence of RIHD has been associated with exposure to IR doses in order of tens of Gray (Gy) [14,15]. However, epidemiological findings have shown that exposure to IR doses of ≤4 Gy has also been associated with RIHD [16,17,18,19]. A typical example is radiation exposures from nuclear accidents, bomb blasts, as well as radiation disasters. Years after the Hiroshima and Nagasaki as well as Chernobyl disasters, some survivors experienced symptoms of RIHD [20]. These side effects negatively affect the supply of blood to heart muscles, leading to an increase in the risk of heart attacks to irradiated persons [16].

The increasing incidence of RIHD has also placed a huge demand for non-invasive cardiovascular imaging modalities, including cardiac computed tomography (CT) scans as well as myocardial perfusion imaging, with single photon emission tomography (SPECT) and positron emission tomography (PET) [21]. The effective radiation doses from these imaging modalities range between 1 to 20 mSv [22]. Although, these radiation doses are considerably low with minimal side effects, the risk of complications due to its overuse could also arise [23]. It was reported that 14%–22% of cardiac imaging tests in the US were inappropriate [24,25]. Hence, the need for effective management of these cardiac imaging techniques.

## 2. Mechanism of Radiation-Induced Heart Disease

IR interacts with cellular components directly and indirectly. Direct effects involve the direct interaction between IR and the DNA. For indirect effects, free radicals such as reactive oxygen species (ROS) and reactive nitrogen species (RNS), which are produced from the radiolysis of water molecules, interact with surrounding DNA molecules to cause damages [26]. IR-induced toxicities, can be in the form of cancerous as well as non-cancerous effects. It has been shown that a linear relationship exists between exposure to IR and carcinogenesis (cancer formation) for clinical radiation doses between 0.15–1.5 Gy [27]. IR-induced DNA damages, which are mostly due to indirect effects, include base damages, crosslinks, single-strand breaks (SSBs), and double-strand breaks (DSBs). Among these damages, which can either occur separately or in combination with another, resulting to complex DNA damage, DSBs are the most severe [28,29]. Although DSBs can be remedied, inadequate repairs could lead to mutations (non-cancerous) and subsequent carcinogenesis [30,31]. While IR damages both normal and cancerous cells, normal cells have greater capacity to repair this damage. This is the underlying principle behind fractionated radiotherapy, in which radiation doses to tumor cells are delivered in multiple small daily doses, to allow for the repopulation and repair of non-cancerous cells, while still providing the toxic effect to the cancerous cells.

IR initiates and modulates inflammatory and immune responses. Factors affecting these responses include radiation type, dose, dose rate, intensity/fractionation, method of delivery, field size, and total cumulative dose [32]. Recent evidences have revealed the roles of complex DNA damage on the strong association between radiation response with inflammatory and immune responses. Complex DNA damage also has a crucial role in IR-induced carcinogenesis [33,34]. Failed or delayed repair of complex DNA damage leads to senescence or cell death including apoptosis, necrosis, and necroptosis. These processes activate the extracellular release of “danger” signals, otherwise known as damage-associated molecular patterns (DAMPs: ATP, short DNAs/RNAs, ROS, heat shock proteins (HSPs), high-mobility group box 1 (HMGB)-1, S100 proteins, etc.) [35]. Subsequently, DAMPs trigger different pattern recognition receptors (PRPs) such as Toll-like receptors (TLRs) and inflammasomes, leading to inflammation and immune-related pathologies.

Another mechanism through which IR induces toxicities is via systemic effect, also known as “non-targeted effect” (NTE). It is a process in which cells or tissues, which do not directly interact with IR, are induced with complex radiation effects including bystander effect, radioadaptive response, and genomic instability. Furthermore, systemic effect has been observed for radiation doses < 1 Gy [35]. Together with immune responses, systemic effect is important in assessing radiation effects as well as reversal to normal physiological state. Moreover, the release of DAMPs from directly irradiated lungs has been shown to impair left ventricular diastolic function in the heart via triggering excessive inflammation and immune response [36].

Radiation-induced side effects can also be early or late, with its severity varying with radiation dose as well as organ type [37]. Early effects such as apoptosis, lymphocyte adhesion and infiltration, vascular permeability, increased endothelial cell swelling, and edema could occur within hours after radiation exposure [38]. However, late effects, including necrosis, organ dysfunction, death, cancer, etc. may arise months to years following exposure [39].

RIHD is a late effect of radiation exposure, with tissue fibrosis as a common endpoint. Fibrosis in the coronary and carotid arteries may disrupt normal blood supply of heart muscles, leading to ischemia and heart failure. Irradiation of tissues could give rise to cell death via apoptosis, necrosis, and mitotic catastrophe, leading to infiltration of inflammatory cells such as mast cells, lymphocytes, and macrophages [40]. Consequently, the chronic secretion of pro-inflammatory and pro-fibrotic cytokines, such as transforming growth factor (TGF-β), tumor necrosis factor-α (TNF-α), and interleukins (IL) (IL-1, IL-4, IL-6, IL-8, IL-10, IL-13) leads to the upregulation of the expressions of some pro-oxidant enzymes, such as NADPH oxidase (NOX), cyclooxygenase-2 (COX-2), and inducible nitric oxide synthase (iNOS) [41,42]. These upregulations are responsible for radiation-induced chronic oxidative stress and fibrosis. Histological findings have also shown that accumulation of inflammatory cells has a crucial role in the onset of chronic oxidative damage, inflammation, and fibrosis, thereby causing variations in the normal features of the heart as well as an increase in the risk of heart attack [43].

Studies have shown that the continuous production of free radicals through immune system-redox interactions has a major role in the late effects of exposure to radiation in heart tissue, which include inflammation and fibrosis [44]. Oxidative stress gives rise to inflammatory mediators, proteases, and adhesion molecules, as well as a reduction in nitric oxide, a vascular protectant that inhibits platelet aggregation and proliferation of vascular smooth muscles [43]. The association between oxidative stress and inflammation has also been linked to nuclear factor-kappa B (NF-κB), a protein complex responsible for the regulation of DNA transcription as well as cellular responses to irradiation [45,46].

## 3. Protection against Radiation-Induced Heart Disease

The rising incidence of RIHD has brought about several approaches towards its prevention. As previously mentioned, most human exposures to IR are due to diagnostic imaging modalities. Therefore, the most basic and important radiation protection strategy is the principle of justification, in which benefits and risks from the use of IR are carefully evaluated. Although, this principle is patient-dependent as well as at physician’s discretion, several guidelines have been published by organizations, such as the European Society of Cardiology (ESC) and the American College of Cardiology Foundation (ACCF), for better implementation of this principle [21,47]. Briefly, the ACCF guideline describes an appropriate cardiovascular imaging procedure as one in which the expected diagnostic information, in addition to clinical judgement, is far more than possible negative effects [48]. The ESC guideline takes into consideration the differences between the various cardiovascular indications. Therefore, while an imaging modality may be inappropriate for a certain clinical indication, it may be appropriate for use in others [49].

Dose-lowering approaches may also be beneficial to patients as well as physicians [50]. Advancements in radiotherapy methods, such as intensity-modulated radiotherapy (IMRT), conformal radiotherapy, image-guided radiotherapy (IGRT), and stereotactic body radiotherapy (SBRT), limit the radiation doses to the irradiated volume, thereby sparing normal tissues during irradiation [51,52]. Another approach—heavy particle radiation—utilizes the Bragg peak phenomenon in reducing dose delivered to healthy tissues [53].

The need to neutralize free radicals, or repair damages induced by these free radicals, forms the basis in the development of radioprotective agents, also known as radioprotectors. An ideal radioprotector should have minimal toxicities and protect normal tissues but not tumor cells [54]. Availability as well as cost effectiveness are also important factors to be considered [55]. A review by Zhang et al. highlighted several natural products including Dan-Hong, San-Yang-Xue-Dai, fermented *Cordyceps sinensis*, *Salvia miltiorrhiza*, curcumin, saponin dioscin, modified Zhi-Gan-Cao-Tang, *platycodon grandiflorum*, flaxseed oil, parsley oil, blueberry anthocyanins-enriched extract, *rubia cordifolia*, Sheng-Mai-San, and zingerone, which have shown protective effects against chemotherapy-induced cardiotoxicity [56].

In present study, we review the various natural products that have been investigated for protection against RIHD. The choice of natural products is because of their low toxicities, availability and cost effectiveness.

## 4. Hesperidin

Hesperidin (hesperetin-7-rhamnoglucoside) is a bioflavonoid found in citrus fruits such as tangerine, orange, lemon, and grape, as well as in plant extracts such as tea and olive oil. The peels of these plants have the highest concentration of hesperidin. It has been used to treat inflammatory as well as allergy diseases [57]. It has also been employed in the treatment of cardiovascular and neurological disorders [58,59]. Studies have shown that hesperidin possesses antimicrobial, anticarcinogenic, decreasing capillary fragility, antioxidant, and anti-inflammatory effects [60]. 

The radioprotective and anti-inflammatory effects of hesperidin against RIHD have been investigated. In a study by Rezaeyan et al., rats, which were treated with 100 mg/kg hesperidin orally for seven consecutive days before thoracic exposure to 18 Gy single dose gamma radiation, showed improvement in survival. Furthermore, reduction of oxidative damage, vascular leakage, inflammation, fibrosis, and infiltration of macrophages, lymphocytes, and mast cells were also observed [61]. Park et al. showed that oral administration of 50 and 100 mg/kg hesperidin doses for seven consecutive days after gamma (γ) irradiation with 5 Gy was effective in ameliorating serum heart disease markers [5]. Pradeep et al. investigated the ability of hesperidin to repair cardiocellular damage following whole-body γ-irradiation [62]. After exposure to a single dose of 5 Gy γ-radiation, they administered 50 and 100 mg/kg hesperidin orally to rats for seven consecutive days. Their results showed that hesperidin treatment post-irradiation significantly ameliorated lipid peroxidation, decreased protein carbonyl levels, reduced cathepsin-D levels, decreased activity of xanthine oxidase, prevented severe destruction to cardiac muscles, and improved glutathione (GSH) content as well as antioxidant level in the heart, in a dose–dependent manner.

## 5. Curcumin

Curcumin (1,7-bis(4-hydroxy-3-methoxyphenyl)-1,6-heptadiene-3,5-dione), also known as diferuloylmethane, is the major natural polyphenol found in the rhizome of *Curcuma longa* (turmeric) as well as other *Curcuma* spp [63]. It is commonly used in traditional Indian and Chinese medicines. It possesses abilities such as antioxidant, anti-inflammatory [64], antimutagenic, antimicrobial [65,66], and anticancer [67,68]. Curcumin’s anti-inflammatory property is responsible for its radioprotective effect, while its anticancer ability makes it a suitable radiosensitizer [69,70,71]. As a free radical scavenger, curcumin can protect against lipid peroxidation and also neutralizes reactive oxygen species (ROS) and reactive nitrogen species (RNS) [72,73].

The radioprotective effect of curcumin on heart tissues was investigated by Kolivand et al. Rats were orally administered 150 mg/kg curcumin for seven consecutive days (one day before and six days after exposure to 15 Gy γ rays). Their results 10 weeks after irradiation showed that curcumin attenuated the upregulation of IL-4 protein and its receptor, IL4Ra1, as well as prevented infiltration of lymphocytes and macrophages. It also attenuated the expression of dual oxidases (Duox1 and Duox2), enzymes responsible for the stabilization of extracellular matrix via oxidative cross-linking [74].

## 6. Melatonin

Melatonin (N-acetyl-5-methoxytryptamine), a hormone largely secreted in the pineal gland, was discovered in 1958 by Aaron Lerner [75]. It has also been observed in various organs and tissues, such as the gastrointestinal tract (GIT) [76,77,78] and some leucocytes [79,80]. It is involved in the circadian regulation of biological and endocrine functions such as mood, sleep, sexual progression and reproduction, immune activities, aging, etc. [81,82,83]. In plants, melatonin can be found in cereals, olive, walnuts, tomatoes, pineapple, ginger, legumes, etc. [84]. The concentration of melatonin in the body differs with time, with its peak secretion at night and lowest during the day [85,86,87,88,89,90]. Melatonin possesses antiapoptotic [91], antioxidant [92], anti-inflammatory [93], tumor sensitizing [94], as well as neuroprotective abilities [95]. Clinical studies have shown promising results in its use in radiotherapy for preventing complications such as dermatitis, and oral mucositis [96,97,98].

Gürses et al. evaluated the ability of melatonin to prevent RIHD. Rats received 50 mg/kg melatonin intraperitoneally 15 min before exposure to a single dose of 18 Gy γ radiation. Histopathological assessments six months after irradiation showed that, compared to the non-treated group, melatonin prevented vasculitis as well as decreased myocyte necrosis and fibrosis [99]. Amelioration of oxidative damage to heart tissues of mice who received intraperitoneal injection of 20 mg/kg melatonin 30 min before exposure to 5 Gy γ rays was observed by Abadi et al. In addition, the activities of superoxide dismutase (SOD) as well as glutathione peroxidase (GPx) were significantly increased [100].

## 7. Selenium

Selenium was discovered by Jons Jacob Berzelius, a Swedish chemist, in 1818 [101]. It is a trace element necessary for the formation of various selenoproteins, which have vital roles in important metabolic processes. Selenoenzymes and selenoproteins are important antioxidant enzymes in the body [102]. Its combination with curcumin was investigated by Amini et al. for its ability to prevent RIHD. Rats received the combination of curcumin (150 mg/kg orally) and selenium- L-methionine (4 mg/kg intraperitoneally), one day before and three consecutive days after exposure to 15 Gy γ rays to their chest areas. Their results 10 weeks post irradiation showed that this combination led to significant reductions in the expressions of IL4Ra1, Duox1, and Duox2, hence implying protection against RIHD via modulation of redox system and chronic oxidative stress [103].

## 8. Caffeic Acid Phenethyl Ester

Caffeic acid phenethyl ester (CAPE) is a natural bioactive compound found in honeybee propolis as well as wine [104], and has been shown to exhibit antioxidant, antimicrobial, anti-inflammatory, antitumor, and immunomodulatory properties [105]. Yilmaz et al. observed that a CAPE concentration of 10 µmol administered intraperitoneally inhibits the production of ROS in human neutrophils as well as xanthine/xanthine oxidase (XO) system [106]. 

In a study by Mansour and Tawfik, they investigated the ability of CAPE to prevent RIHD. CAPE dose of 10 µmol/kg was administered intraperitoneally to rats for seven consecutive days after exposure to 7 Gy γ radiation. This led to significant reductions in the activities of malondialdehyde (MDA), xanthine oxidase (XO), and adenosine deaminase (ADA), as well as significant increase in the levels of total nitrate/nitrite (NO(x)) and SOD in heart tissue. Furthermore, serum lipid levels and cardiac enzymes were restored [107].

## 9. Black Grape Juice

Black grape juice (BGJ) is rich in resveratrol and quercetin with potent anticancer abilities [108]. These antitumor effects have also been observed in several studies [109,110]. It has been shown to prevent RIHD in rats. Each animal was treated 2 mL/day of BGJ via gavage one week before and four days after whole body exposure to 6 Gy γ radiation. A significant reduction in MDA was observed for the irradiated group who were administered BGJ, indicating its positive effect against oxidative damage to the heart [111].

## 10. Zingerone

Zingerone [4-(4-hydroxy-3-methoxyphenyl)-2-butan-2-one], a natural polyphenol, is one of the components of ginger (commonly used as herb or spice). Zingerone, in addition to gingerols, shogaols and paradols, constitute the pungent sensation exhibited in ginger [112]. Studies have shown that zingerone possesses antioxidant [113,114], anti-inflammatory [115], anticancer [116], and antimicrobial [117] effects, as well as protects against radiation-induced cytotoxicity [118] and genotoxicity [119].

The cardioprotective effect of zingerone against IR-induced oxidative stress, inflammation, and apoptosis was investigated by Soliman et al. [120]. Daily doses (25 mg/kg) of zingerone were administered to rats via intragastric intubation for three weeks before exposure to a single dose of 6 Gy γ-radiation to whole body. Their results showed that pre-treatment with zingerone led to significant reduction in abnormalities to heart tissues as well as increased radiation-induced cardiotoxicity indexes, including serum lactate dehydrogenase and creatine kinase-MB activities, in addition to plasma cardiac troponin T and B-natriuretic peptide. Furthermore, oxidative stress was ameliorated, as indicated by a significant reduction in malondialdehyde (MDA) level, as well as significant increase in both GSH and catalase activities. Moreover, zingerone administration led to a reduction in inflammatory markers, such as serum level of tumor necrosis factor-alpha, cardiac myeloperoxidase activity, and cyclooxygenase-2 protein expression. Finally, it attenuated the decrease in the activities of mitochondrial complexes, and mitigated the effects of elevated caspase-3 gene expression and prominent nuclear DNA fragmentation.

## 11. Sheng-Mai-San

Sheng-Mai-San (SMS) is a common herbal formula in traditional Chinese medicine. Its constituents include Panax ginseng, Ophiopogon japonicus, and Schisandra chinensis. SMS has been shown to possess antioxidant properties [121,122]. Epidemiological findings have revealed the use of SMS in the treatment of various diseases for over 800 years [123]. Initially, SMS was used in treating deficiencies of qi and yin syndromes of CVDs, which are associated with symptoms of fatigue, weakness, heat stroke, thirst, etc. [124]. Presently, SMS has wide range of applications in the treatments of cancer [125], myocardial ischemia [126], myocardial infarction [127], as well as protects against right ventricular dysfunction [123], myocardial injury [121], and renal ischemia [128]. Its long-term administration has also been shown to improve quality of life of patients with pulmonary heart diseases [129,130].

A clinical trial by Lo et al. was conducted to evaluate the therapeutic efficacy of SMS in the treatment of adult cancer patients (≥ 18 years) undergoing radiotherapy [131]. The patients were administered eight 0.5 mg SMS capsules orally, thrice a day for four weeks. Using the European Organization for Research and Treatment of Cancer (EORTC) Quality of Life questionnaire (QOL-C30), their results showed that treatment with SMS alleviated radiotherapy-induced side effects as well as improved heart function.

The mechanisms by which these reviewed natural products protect against RIHD are summarized in Table 1.

## 12. Conclusions

The rising incidence of cardiovascular diseases is of great concern. It has been estimated that by 2030 the annual mortality from CVDs will hit more than 23.3 million people [132]. Therefore, concerted efforts should be intensified to ensure that the risk factors of CVDs are controlled or prevented in order to minimize the risks of CVDs. This review was centered on one of the common risk factors of CVDs: exposure to IR. The increasing use of IR for cancer treatment, as well as for diagnostic purposes, may give rise to RIHD if adequate protective measures are not in place. Experimental evidences have shown that the reviewed natural products have the potentials for possible protection against RIHD. Their potentials will still require further evaluation by conducting more experimental studies to confirm previous results. Furthermore, no toxicities from the administration of these natural products were observed, thereby dispelling safety concerns in future clinical trials. However, the efficacy of these products is yet to be examined in clinical trials, except for Sheng-Mai-San (NCT number: NCT01580358). Hence, we suggest future clinical trials using these reviewed natural products to examine if the administered experimental doses would be realistic in a clinical situation, as well as for more insights due to possible differences between experimental outcomes involving cells or animals and those from humans. In addition, the molecular mechanisms through which their radioprotection is achieved should be further elucidated. This will go a long way in reducing the incidence of RIHD as well as to improve therapeutic index of radiotherapy.

## Figures and Tables

**Table 1:** Natural products and mechanisms of protection against radiation-induced heart diseases (RIHD).

Natural Product	Mechanism against RIHD
Hesperidin	Antioxidant and anti-inflammatory effects.
Curcumin	Anti-inflammatory
Melatonin	Antiapoptotic, antioxidant and anti-inflammatory effects.
Selenium	Antioxidant effect
Caffeic acid phenethyl ester (CAPE)	Antioxidant and anti-inflammatory effects.
Black grape juice (BGJ)	Antioxidant effect
Zingerone	Antioxidant and anti-inflammatory effects.
Sheng-Mai-San (SMS)	Antioxidant effect
